# Supplementary material for: The mRNA-bound Proteome of Leishmania mexicana: Novel Genetic Insight into an Ancient Parasite
Source: Mol Cell Proteomics. 2019 Apr 4;18(7):1271–84. doi: 10.1074/mcp.RA118.001307 (PMC6601212; doi:10.1074/mcp.RA118.001307)
Supplement: Supplemental Figures [file 142857_1_supp_289999_pn77xh.pdf]

Supporting information captions

Figure S1. XL and nonXL RBPomes are distinct from whole proteomes and nearly identical. A. PCA analyses of all Proteome data. Whole proteomes segregate from RBPomes regardless of lifecycle stage. B. Venn Diagram of XL vs nonXL RBPomes without filtering for differential enrichment. Close overlap reveals isolated XL and nonXL RBPome protein identities are nearly identical.

Figure S2. Venn Diagram comparison of enriched proteomes across the *Leishmania* lifecycle. (A) Protein enrichment analyses compare each lifecycle stage of the WC, XL and nonXL proteomes. Proteomes of each lifecycle stage were filtered to select highly enriched proteins with mean intensity values across each of the 3 replicates ≥ 10^6. These enrichment-selected proteins were used to create Venn diagrams in Python 2.7 using Matplotlib v.1.5.3 and Matplotlib-Venn package version 0.11.5. (B) Complementary Venn diagrams comparing the WC, XL and nonXL proteomes of each lifecycle stage.

Figure S3. Relative RBP protein and mRNA expression through lifecycle progression. Relative expression of endogenously-tagged LmxM.34.2200 (DRBD2), LmxM.21.1552 (SUB2), LmxM.28.0825 (RBP16), LmxM.04.1170 (DRBD3) and LmxM.25.0490 (UBP1) is shown via anti-HA western blot and qRTPCR analyses. Levels of each were quantified relative to NMT protein and transcript expression [26].

Table S1. Relative intensity of filtered proteomes. Accession numbers and relative intensities (percentage) of RBPome proteins isolated in XL, nonXL and WC proteomes filtered for triplicate-consistent, high quality reads with at least 2 unique peptides are provided. Red to Blue color gradient is indicative of relative intensity values. Intensity sum for

each row = 100%. Relative Percent Intensity values were calculated by expressing normalized intensity values for each protein as the percentage of the sum of normalized intensity values among all samples. Q value represents significance of intensity shift between different conditions of each triplicate. Presented q-values are Hochberg and Benjamini multiple-test corrected false discovery rates calculated from Progenesis QI ANOVA tests for significant difference between sample groupings. Q-values <0.05 are highlighted in green. Identification descriptions are approximate and predominantly homology-based as the overwhelming majority of factors are uncharacterized in this system.

Table S2. Label-free Quantification Summary. Table of Progenesis QI-derived label-free quantified proteins detailing relative raw protein abundances observed among all samples. Columns headed Normalized Intensity list summed MS^1 peak areas extracted from non-conflicting peptides post normalisation against total ion intensity. Statistical analyses are as in Table S1.

Table S3. Primers used in experimental procedures.

Figure S1.

A. PCA of all samples

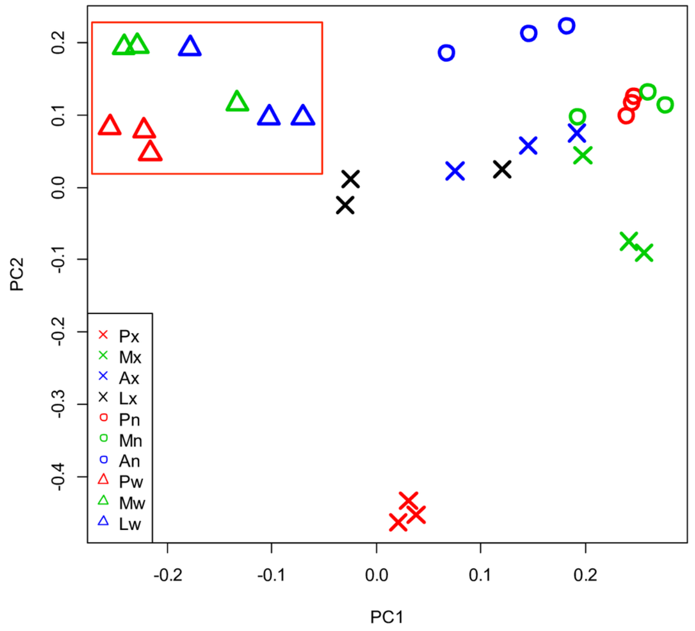

B. XL vs noXL PCF RBPomes
(intensity > 0)

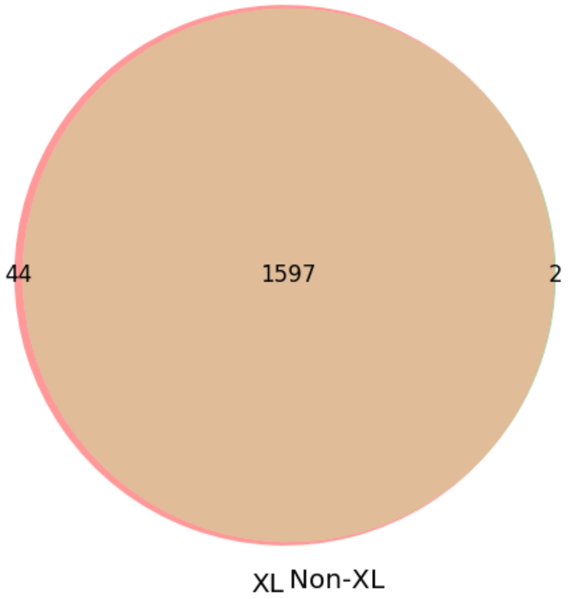

Figure S2.

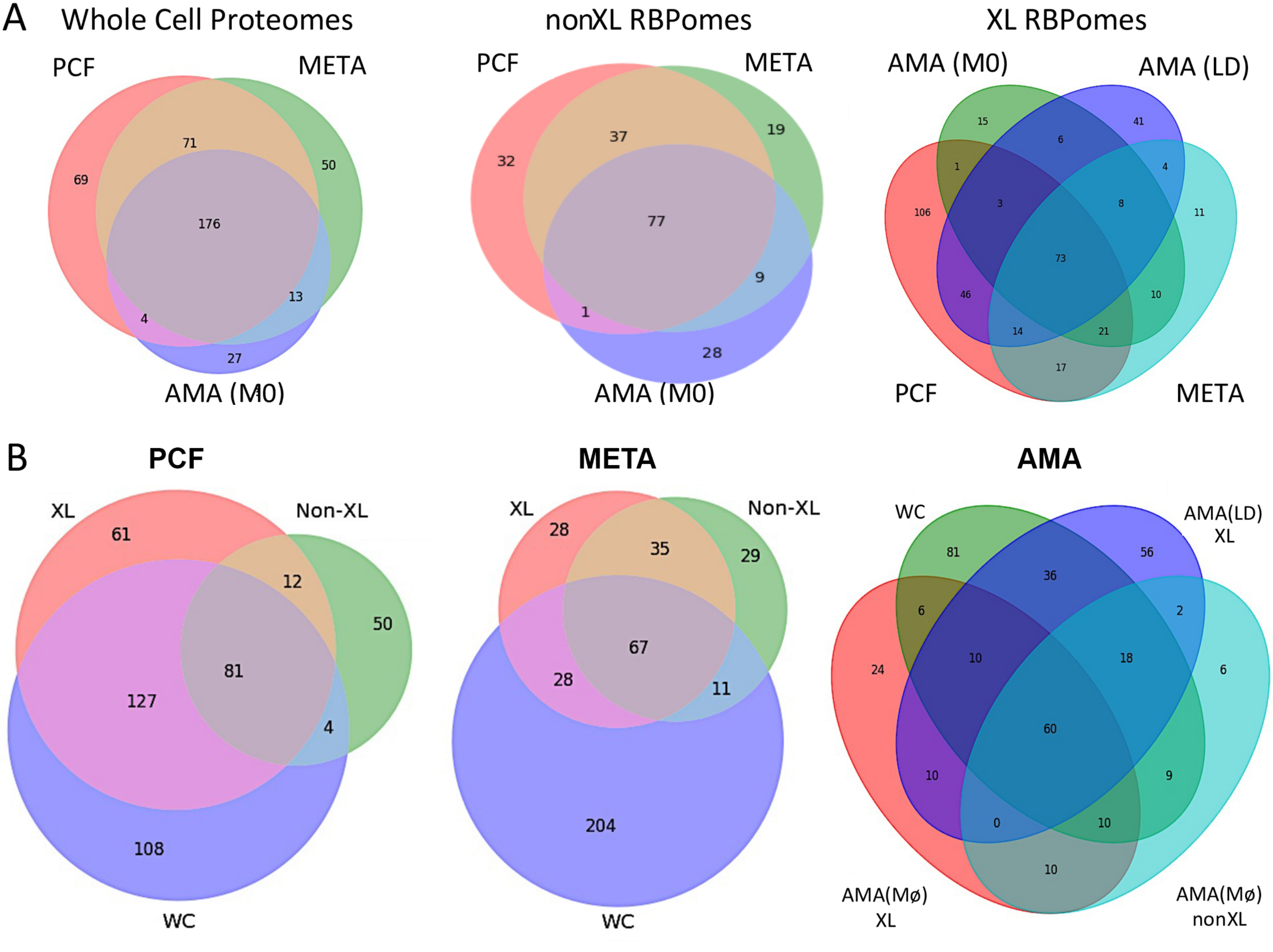
A
Whole Cell Proteomes
PCF
META
AMA (M0)
71
69
50
176
13
4
27
nonXL RBPomes
PCF
META
AMA (M0)
37
32
19
77
9
1
28
XL RBPomes
AMA (M0)
AMA (LD)
PCF
META
15
41
6
1
4
106
3
8
11
73
46
10
14
21
17
B
PCF
XL
Non-XL
WC
61
12
50
81
127
4
108
META
XL
Non-XL
WC
28
35
29
67
28
11
204
AMA
WC
AMA(LD) XL
AMA(Mø) XL
AMA(Mø) nonXL
81
56
36
6
2
24
10
18
6
60
10
9
0
10
10

Figure S3.

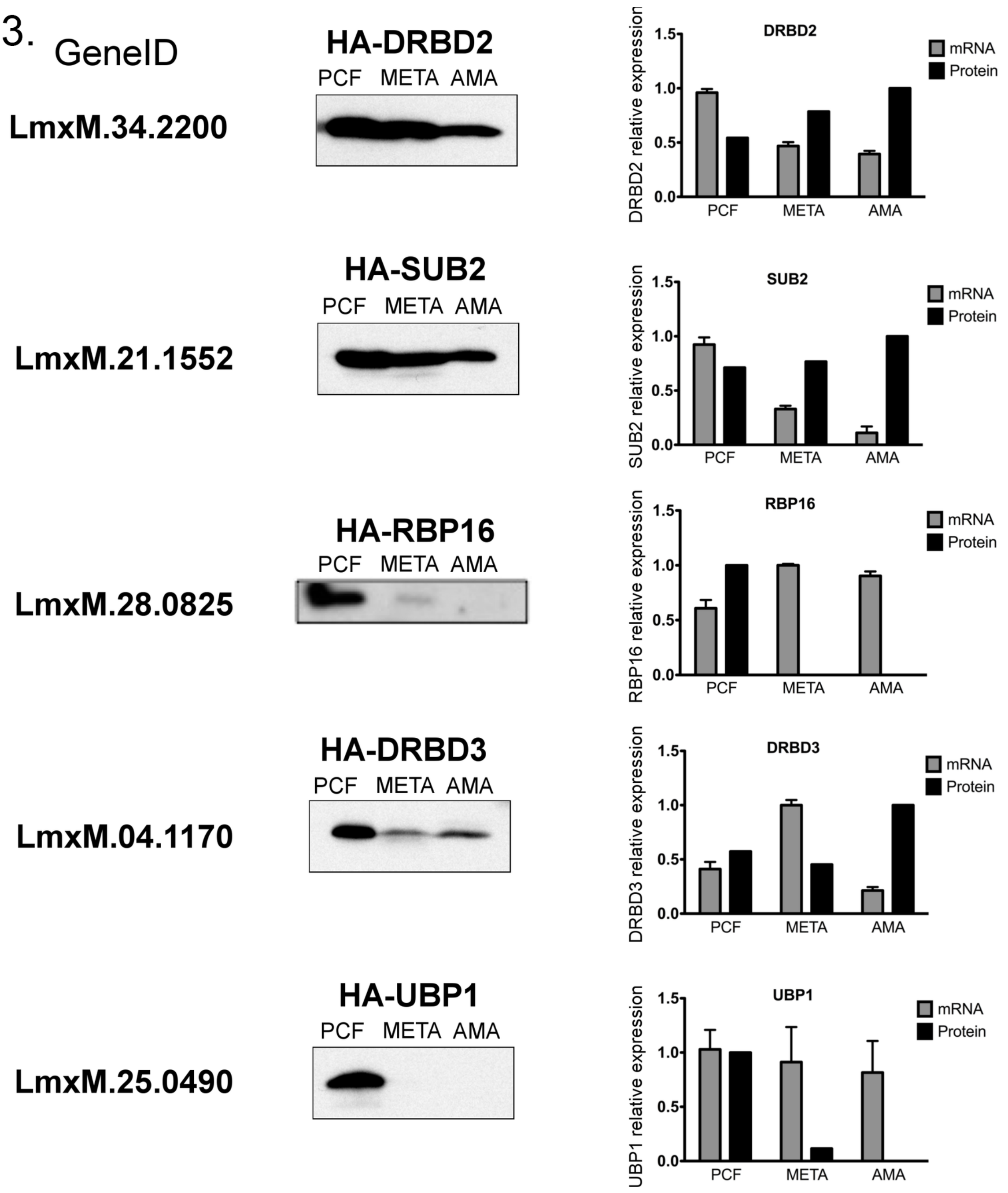
GeneID
LmxM.34.2200
HA-DRBD2
PCF META AMA
DRBD2
DRBD2 relative expression
1.5
1.0
0.5
0.0
PCF
META
AMA
mRNA
Protein
LmxM.21.1552
HA-SUB2
PCF META AMA
SUB2
SUB2 relative expression
LmxM.28.0825
HA-RBP16
PCF META AMA
RBP16
RBP16 relative expression
LmxM.04.1170
HA-DRBD3
PCF META AMA
DRBD3
DRBD3 relative expression
LmxM.25.0490
HA-UBP1
PCF META AMA
UBP1
UBP1 relative expression
